# School participation of students with disabilities in Spain: profile, supports and barriers

**DOI:** 10.3389/fpsyt.2026.1783581

**Published:** 2026-03-26

**Authors:** María Gómez-Vela, Eva González Ortega, María Begoña Orgaz Baz, Isabel Vicario-Molina

**Affiliations:** 1 Department of Personality, Assessment and Psychological Treatment, University of Salamanca, Salamanca, Spain; 2 Department of Developmental and Educational Psychology, University of Salamanca, Salamanca, Spain; 3 Department of Basic Psychology, Psychobiology and Methodology of Behavioral Sciences, University of Salamanca, Salamanca, Spain

**Keywords:** child, disability, school participation, environment, barriers, supports

## Abstract

**Introduction:**

School participation is central to learning, development, and inclusion. Within international efforts to identify environmental barriers to participation, this study aimed to analyze the school participation profile of students aged 5–17 years in Spain—with and without disabilities and other special educational needs (SEN)—and to identify environmental factors that support or hinder their participation.

**Method:**

Parents of 415 Spanish students aged 5–17 years completed the Spanish version of the Participation and Environment Measure for Children and Youth: PEM-CY. Group differences between children with (n=142) and without (n=273) disabilities were tested.

**Results:**

Students with disabilities/SEN participated less often and with lower involvement across all school activities, with significant differences in “Classroom activities” and “Getting together with peers outside of class.” Parents of children with disabilities reported that cognitive, behavioral, and social demands commonly hinder participation. Reported resource unavailability ranged from 10%–38% (adapted transportation, financial resources, information, technical/communication aids, and support services tailored to students’ needs and interests). Conversely, 44.4%–72.8% of parents indicated that physical layout, sensory qualities, safety conditions, staff attitudes, and peer relationships usually supported participation.

**Discussion:**

Despite inclusive-education mandates, findings indicate persistent participation and engagement inequities for students with disabilities and other special needs in Spanish schools. Parents point to environmental conditions—support services tailored to students’ needs and interests, teacher training to individualize these supports, and a culture of collaboration among teachers and between teachers, families, and children with special needs themselves—as key elements in ensuring meaningful, equitable, and full participation in school life.

## 1 Introduction

Participation, defined as “an individual’s involvement in life situations” (1), has a positive impact on children’s health, development, and well-being (2, 3) and supports positive trajectories into adulthood (4). Through participation in daily life activities at home, at school, or in the community, children acquire skills, improve physical and mental health, gain emotional wellbeing, develop friendships and social networks, and become self-determined (5, 6).

Participation in school refers to attendance and, especially, to engagement (7, 8). It includes everyday life situations or activities that are required or desired to fulfil the role of the school pupil within or around the school context (9). It encompasses a range of possible roles and activities, including classroom activity, schoolwork, events, trips, teams, clubs, and relationships (9, 10). It refers to unstructured activities (e.g. friendships, play), organized activities (e.g. sports), classroom-based activities (e.g. group work), and engagement in desirable roles within the school (11, 12). It is related to three core elements of inclusive education (13): 1) sharing common classrooms and school places and the possibility to learn together, 2) recognizing the feeling of belonging to a group, and 3) being involved in educational decision-making.

At school, participating in a wide variety of activities provides children with opportunities to enhance their independence and social interaction with diverse peer groups (14, 15), which is essential for fostering skill development and academic progress (7). Furthermore, it contributes to reducing school dropouts, facilitating career choice (16, 17), promoting social involvement in young adulthood (11, 17, 18) and increasing well-being (19) and quality of life (20–22).

The participation of children with disabilities and other special educational needs (SEN) in the daily life of the school is crucial for skills development and integration in society (23), and therefore, it is an essential element of inclusive education, both as a condition and an indicator for its effectiveness (8, 9, 24). Its restriction may negatively affect the development and well-being of these students (2, 21, 25, 26).

Moreover, participation is a right of persons with disabilities (27 and a key outcome of rehabilitation interventions (8, 28, 29). The Convention on the Rights of Persons with Disabilities obligates States Parties to provide equal opportunity for students with disabilities to participate in all aspects of the educational experience (27, 30) and requires them to provide environmental supports to ensure their successful school participation (31).

Unfortunately, nonetheless, much research has reported that the participation of children with disabilities is restricted in the school context (4, 31–33), particularly when being compared to peers without impairments. Students with disabilities and other SEN show lower school attendance (34, 35), participate less in structured and unstructured activities (36, 37), and experience reduced interaction and playground participation (38, 39). In addition, they show less engagement in the wider school world, including clubs and organizations (35, 37, 40, 41), decreased frequency of interactions with peers outside of class (36, 42) and less desirable roles within the school (12).

For all the above-cited reasons, UNESCO (43) recommended in 2020 to use evidence to pinpoint context-specific barriers that limit learners’ participation and progress. Indeed, school participation of children with disabilities may be negatively and positively influenced by both individual and environmental factors. Frequently mentioned environmental *barriers* to participation include the physical design of schools (4, 33, 34), lack of needed services as well as services that are not tailored to the students’ needs and interests (4, 31, 32, 34, 44), limited understanding of disability and insufficient training of teachers to attend to diversity and to adapt the tasks to students’ characteristics (34), poor fit between functional limitation of children with disabilities and activity demands (31, 37), lack of time to collaborate with other teachers (33), lack of tools and adapted materials (39, 45, 46), negative attitudes of some members of the educational community (47, 48), and lack of programs and services which support school participation (31, 37).

On the other hand, environmental *facilitators* for school participation include teacher-students positive interactions (49), use of Universal Design for Learning (UDL) and augmentative or alternative communication systems (AACs) (33), social support of family and classmates (2, 37, 42, 50), accessible transportation and after school activities open to all (31, 42, 51, 52), and collaborative partnerships between families, schools and outside organizations (14, 33, 34).

The literature also shows evidence of *individual* influencing factors, including the type of impairment (34), student’s socioeconomic status (4, 10), age and grade level (2, 44), autonomy (40), functional abilities (2, 52, 53), locus of control (49), preferences, interest and motivations (9), presence of pain and fatigue, resilience and social and solving-problems skills (39), or satisfaction associated with activities (54). However, most studies emphasize the relevance of environmental factors over personal factors in school participation (9, 24, 33, 55, 56).

In Spain, the Law on the Rights of Persons with Disabilities and their Social Inclusion (57) contemplates the principle of full participation and inclusion and the realization of reasonable adjustments of the physical, social, and attitudinal environment to facilitate the participation of persons with disabilities under equal conditions. Likewise, the current Spanish legislation on education (58) indicates that students with special educational needs are those who face barriers that limit their access, presence, and participation in education, and establishes the obligation to develop actions to eliminate such barriers so that all students have equal access to inclusive education.

In order to fulfill these mandates and prior to environment-based interventions, it is essential to further analyze the participation of these children at school as well as the environmental supports and barriers through the use of valid and reliable measures (4, 59). A recent review highlights the need to identify contextual determinants to promote it effectively (56). Moreover, the evidence on the factors that support or hinder the school participation of children and youth with disabilities is limited in some countries. To our knowledge, there is no evidence in Spain on these topics. Therefore, the aims of this study were: 1) to analyze the school participation profile of Spanish students from 5 to 17 years with and without disabilities and other educational needs, and 2) to identify environmental factors that support or hinder their participation. The ultimate goal was to offer orientations to design best practices to improve the participation of all students at school.

## 2 Materials and methods

### 2.1 Participants

Participants were considered eligible if they met the following criteria: a) being parents or legal guardians of children or youths (from 5 to 17 years old) with or without disabilities or other SEN, b) being able to read Spanish, c) residing in Spain. The sample of this study was composed of 415 parents (**Table 1**), the majority of them mothers (88.4%) from nuclear families, of children or youth with (34.1%) and without (65.9%) disabilities. A large proportion of both parents had higher vocational or university education and were employed. The mean age of the children was 10.51 years (SD = 3.32) and 54.6% were boys. The majority (95%) of them attended mainstream schools. The most commonly reported diagnosis included health conditions followed by intellectual disability, delayed educational system incorporation or low school performance and learning disabilities (**Table 2**).

**Table 1:** Sociodemographic characteristics of the participating parents and their children.

Sociodemographic characteristics	Total sample (*n* = 415)
	*n* (%)
Respondent	
Mother	367 (88.4)
Father	48 (11.6)
Father’s educational level	
No school certificate	6 (1.4)
Primary school	94 (23.2)
Secondary school or secondary vocational education	97 (24.0)
Higher vocational education	52 (12.8)
University	156 (38.6)
Mother’s educational level	
No school certificate	2 (0.5)
Primary school	35 (8.4)
Secondary school or secondary vocational education	74 (17.8)
Higher vocational education	61 (14.7)
University	243 (58.6)
Father’s employment status	
Employed	325 (92.1)
Unemployed/retired	28 (7.9)
Mother’s employment status	
Employed	278 (77.1)
Unemployed/retired	83 (22.9)
C*hild gender* Male Female	225 (54.2) 190 (45.8)
*Child age* 5–11 years (Primary student) 12–17 years (Secondary student)	239 (57.6) 143 (34.4)
Child with disability/diagnoses	
No	273 (65.9)
Yes	142 (34.1)

**Table 2:** Parent-reported diagnoses of students with disabilities/SEN.

Disability/SEN	Total sample (*n* = 142)
	*n* (%)
Health conditions (e.g., chronic health problems, frequent hospitalization), Intellectual disability Delayed educational system incorporation or low school performance Learning disabilities Autism spectrum disorder Physical disability Attention deficit hyperactivity disorder Hearing impairment Giftedness Vision impairment Developmental delay Speech/language impairment Emotional impairment	32 (22.4) 23 (15.8) 19 (13.7) 17 (12.0) 12 (8.3) 9 (6.4) 9 (6.4) 8 (5.4) 5 (2.2) 3 (2.1) 2 (1.5) 2 (1.5) 1 (0.8)

### 2.2 Instruments

The Spanish version of the Participation and Environment Measure for Children and Youth (PEM-CY) was used [Blinded for peer review]. This parent-report questionnaire evaluates children’s participation and environment at home, school and community settings (36, 60). The school section assesses:

- Children’s *participation profile* (5 items) in broad types of activities (classroom activities; field trips and school events; school-sponsored teams, clubs and organizations; getting together with peers outside of class; special roles at school) considering three dimensions: frequency (from never = 0 to daily = 7), involvement (from minimally involved = 1 to very involved = 5) and desire for change (yes/no).
- *Supportiveness of the school environment*: whether certain factors (n = 10) function as barriers or facilitators to participation (i.e., classroom space, playground, and other school facilities; comfort of the school and its spaces; external weather conditions; physical and cognitive demands required for daily school activities; social/communication and self-regulation skills required for daily school activities; attitudes and behaviors of teachers and school staff toward the child; child’s relationships with peers; school safety) with three response options: usually helps, usually hinders, neither helps nor hinders.
- *Availability of resources:* the extent to which support resources (n = 8) are available (i.e., access to adapted transport to get to school; programs and specialized services; school plans and rules; products and technology -pictograms, adapted furniture and materials, adapted sports equipment, technical aids, easy-to-read materials; information; time and money to support the child’s participation in school activities) with three response options: usually yes, usually no, sometimes yes and sometimes no.

This instrument is a valid, reliable measure for assessing the participation of children and adolescents (5–17 years), with or without disabilities (36, 60). The validity and reliability indices of the Spanish version of the scale are, in general, adequate. Internal consistency ranged from acceptable to excellent across participation and environment scales (α = .58–.97). At school, Cronbach’s alpha was.60 for Frequency, .79 for Involvement, and.89 for Overall Environmental Supports [Blinded for peer review]. For the current study, reliability indices ranged from acceptable to excellent: Cronbach’s alpha was.66 for Frequency, .76 for Involvement, and.89 for Overall Environmental Supports.

In addition, a sociodemographic questionnaire was designed to obtain information about child and family characteristics (**Table 1**), including whether the child had a disability/SEN or a health condition, and the specific condition when applicable. The student’s tutor/teacher confirmed whether the student had SEN and specified the type of SEN. The school section included questions about factors such as the type of school (mainstream, special education, or combined), where the child typically receives support (e.g., in a special/support classroom, within the general classroom, or both), the availability of infrastructure, services, and resources (e.g., adapted transport; elevators/ramps; assistive technologies for mobility and communication), teacher training for inclusive education, physical, emotional and task completion support, and the school’s attitudes and social climate.

### 2.3 Procedure

Ethics approval for the study was obtained from [blinded for peer review]. Participants were recruited through convenience sampling via primary and secondary schools as well as associations of families of children with disabilities located across all geographical regions of Spain. In addition, a snowball sampling strategy was employed to reach the largest possible number of participants. School principals and association representatives were provided with detailed information on the study via email and telephone. Parents of children and youth aged 5–17 with and without disabilities were asked to sign the informed consent form and voluntarily complete the PEM-CY if they met the abovementioned inclusion criteria.

### 2.4 Data analysis

Descriptive statistics were employed to summarize participants’ characteristics and, separately, perceptions of participation (frequency, involvement, and desire for change) and of the environment (supportiveness, availability of resources). Means and standard deviations were reported for continuous variables, and percentages for categorical variables.

Given the wide age range (5–17 years), analyses were stratified by school stage (primary: 5–11; secondary: 12–17) to examine potential age-related differences in participation outcomes. Thus, analyses compared students with and without disabilities on each participation item for the two age groups, by using t-tests for independent samples in the case of frequency and involvement, and chi-squared analyses for frequency recategorized as never *vs*. ever participates, and desire for change. Significant differences between stages and their direction are reported where applicable.

The analysis of environment scores did not include the comparison of students with and without disabilities because more than 70% of parents of students without diagnosis reported “Not applicable. My child does not have disabilities”. Therefore, primary and secondary students were compared for the disability group. Chi-squared analyses were carried out for each item as regards supportiveness and availability of resources at school. Analyses were conducted with SPSS-26, setting the level of significance at.01.

## 3 Results

### 3.1 School participation profile

The mean frequency of participation was lower for students with disabilities/SEN than for students without them in all types of school activities, except for “Field trips and school events”. However, these differences were only significant for “Classroom activities” and “Getting together with peers outside of class”. This pattern was observed for both primary and secondary students, that is, there were no significant differences according to age group (**Table 3**).

**Table 3:** School participation patterns of primary and secondary students with and without disabilities/SEN.

Primary school students									
Items	Frequency		Never participates		Involvement			Desires change	
	Disability M (SD)	No disability M (SD)	Disability % (n)	No disability % (n)	Disability M (SD)	No disability M (SD)	Disability % (n)		No disability %(n)
1. Classroom activities	5.54 (2.19)	6.20 (1.48)*	7.1 (6)	2.0 (3)	3.46 (1.24)	4.44 (0.85)*	61.2 (52)		26.1 (40)*
2. Field trips and school events	2.67 (1.91)	2.20 (1.94)	10.8 (9)	19.6 (30)	3.80 (1.24)	4.23 (1.14)*	60.0 (51)		38.8 (58)*
3. School sponsored teams, clubs and organizations	2.51 (2.81)	3.14 (2.80)	48.8 (41)	36.8 (56)	2.67 (1.59)	3.65 (1.40)*	68.9 (58)		35.5 (54)*
4. Getting together with peers outside of class	5.82 (2.26)	6.60 (1.06)*	8.33 (7)	0.0 (0)*	3.79 (1.27)	4.70 (0.59)*	57.6 (49)		15.6 (24)*
5. Special roles at school	0.54 (0.50)	0.60 (0.49)	45.9 (39)	40.1 (61)	2.67 (1.56)	3.48 (1.52)*	61.2 (52)		31.6 (49)*

Secondary school students									
Items	Frequency			Never participates		Involvement		Desires change	
	Disability M (SD)	No disability M (SD)		Disability % (n)	No disability % (n)	Disability M (SD)	No disability M (SD)	Disability % (n)	No disability %(n)
1. Classroom activities	4.78 (2.31)		5.73 (1.81)*	8.7 (4)	2.1 (2)	3.51 (1.27)	4.17 (0.83)	66.0 (31)	38.5 (37)*
2. Field trips and school events	1.85 (2.04)		1.72 (1.91)	34.8 (16)	32.6 (31)	3.28 (1.50)	3.76 (1.25)	82.6 (38)	50.0 (47)*
3. School sponsored teams, clubs and organizations	1.96 (2.60)		2.18 (2.64)	58.6 (28)	48.9 (46)	2.48 (1.66)	3.01 (1.55)	82.6 (38)	52.1 (49)*
4. Getting together with Peers outside of class	5.72 (2.39)		6.71 (1.01)*	12.8 (6)	1.1 (1)*	3.85 (1.35)	4.58 (0.78)*	59.6 (28)	17.0 (16)*
5. Special roles at school	1.40 (2.37)		1.81 (2.50)	68.1 (32)	53.2 (50)	2.04 (1.49)	2.77 (1.46)*	67.4 (29)	49.5 (46)*

*p <.01.

In the five types of activities, a higher percentage of parents of students with disabilities/SEN reported that their children never participate, except for “Field trips and school events” among primary students. Significant differences were only observed in “Getting together with peers outside of class” for both primary and secondary students (**Table 3**).

Parents of students with disabilities in both age groups reported lower levels of involvement across all areas of school participation. Among primary students, the differences were significant for the five types of activities, whereas among secondary students, only for “Getting together with peers outside of class” and “Special roles at school” (**Table 3**).

Finally, a significantly higher percentage of parents of students with disabilities/SEN, regardless of age group, desired some type of change in their child’s participation in all school activities (**Table 3**).

### 3.2 Environmental barriers and facilitators

As for the supportiveness of the school environment for students with disabilities/SEN, a higher percentage of parents considered that the diverse types of environmental demands (cognitive, behavioral, social, physical) usually hindered participation. In contrast, a wider percentage of parents reported that the attitudes of teachers and staff and relationships with peers, safety conditions, physical layout, and sensory qualities usually helped. Physical demands for secondary students and weather conditions were more frequently regarded to neither help nor hinder participation. There were no significant differences in the distribution of response categories between parents of primary *vs*. secondary students with disabilities (**Table 4**). This suggests that perceived environmental barriers and facilitators were largely similar across school stages in this sample.

**Table 4:** Perceived supportiveness of the school environment for students with disabilities/SEN: comparison of primary and secondary students.

Items	Usually hinders		Neither helps nor hinders		Usually helps	
	Primary % (n)	Secondary % (n)	Primary % (n)	Secondary % (n)	Primary % (n)	Secondary % (n)
1. Physical layout	8.8 (7)	2.2 (1)	43.8 (35)	47.8 (22)	47.5 (38)	50.0 (23)
2. Sensory qualities	13.6 (11)	11.1 (5)	35.8 (29)	44.4 (20)	50.6 (41)	44.4 (20)
3. Weather conditions	37.0 (30)	20.0 (9)	51.9 (42)	60.0 (27)	11.1 (9)	20.0 (9)
4. Physical demands	40.0 (32)	23.9 (11)	32.5 (26)	45.7 (21)	27.5 (22)	30.4 (14)
5. Cognitive demands	55.6 (45)	52.2 (24)	16.1 (13)	19.6 (9)	28.4 (23)	28.3 (13)
6. Social demands	44.4 (36)	41.3 (19)	17.3 (14)	30.4 (14)	38.3 (31)	28.3 (13)
7. Behavioral demands	46.2 (37)	37.8 (17)	18.8 (15)	26.7 (12)	35.0 (28)	35.6 (16)
8. Attitudes of teachers/staff	12.4 (10)	13.3 (6)	14.8 (12)	20.0 (9)	72.8 (59)	66.7 (30)
9. Relationships with peers	20.0 (16)	21.7 (10)	11.3 (9)	13.0 (6)	68.8 (55)	65.2 (30)
10. Safety	6.2 (5)	8.9 (4)	34.6 (28)	33.3 (15)	59.3 (48)	57.8 (26)

Finally, most parents of students with disabilities/SEN reported that their children usually had available all resources considered to support participation. Again, there were no significant differences between age groups, except for two resources, public transportation and information, which were considered available by a significantly higher percentage of parents of secondary students with disabilities/SEN. However, it is worth noting that 13 to 38% of parents perceived that the following resources were usually not available: adapted transportation (especially for primary students), money, information, supplies and technology (e.g., pictograms, adapted furniture and/or sports equipment, technical aids, easy-to-read materials, etc.), programs and services (special teacher, speech-language therapist, physiotherapist, etc.) and policies and procedures. No significant differences were observed in the distribution of response categories between parents of primary and secondary students with disabilities (**Table 5**).

**Table 5:** Perceived availability of resources for students with disabilities/SEN: comparison of primary and secondary students.

Items	Usually no	Sometimes yes/ sometimes no				Usually yes
	Primary % (n)	Secondary % (n)	Primary % (n)	Secondary % (n)	Primary % (n)	Secondary % (n)
1. Personal transportation	15.2 (10)	20.0 (7)	18.2 (12)	8.6 (3)	66.7 (44)	71.4 (25)
2. Public transportation	37.9 (25)	21.9 (7)	13.6 (9)	9.4 (3)	48.5 (32)	68.8 (22)
3. Programs and services	6.7 (5)	15.0 (6)	24.0 (18)	17.5 (7)	69.3 (52)	67.5 (27)
4. Policies and procedures	6.9 (5)	13.2 (5)	15.1 (11)	10.5 (4)	78.1 (57)	76.3 (29)
5. Supplies	6.9 (5)	15.4 (6)	26.0 (19)	28.2 (11)	67.1 (49)	56.4 (22)
6. Information	12.2 (9)	22.5 (9)	29.7 (22)	10.0 (4)	58.1 (43)	67.5 (27)
7. Time	1.4 (1)	10.5 (4)	34.3 (25)	26.3 (10)	64.4 (47)	63.2 (24)
8. Money	20.3 (15)	25.6 (10)	36.5 (27)	25.6 (10)	43.2 (32)	48.7 (19)

## 4 Discussion

The aims of this study were to analyze the school participation profile of Spanish students from 5 to 17 years with and without disabilities and other SEN, and to identify environmental factors that support or hinder their participation.

Regarding the first objective, the school participation profile of the participants does not differ from that reported in most studies published recently in other countries: students with disabilities/SEN participate less and are less involved in all the assessed school activities, as compared to their peers without these characteristics. Therefore, despite international recommendations and the legislation above mentioned (27, 58), these students’ right to participate in all aspects of the educational experience is not being guaranteed nor upheld. Moreover, since participation is an essential element of inclusive education (8, 9, 24), the observed restrictions are seriously compromising the assurance and effectiveness of that inclusion.

Specifically, it was found that students with disabilities/SEN participate significantly less frequently in “Classroom activities”, in line with prior findings (37, 44). In more than half of the cases (51.4%), these students receive support from specialist staff (e.g., special-education teachers, speech-language therapists) in separate classrooms at various times during the school day, which reduces the frequency with which they participate in activities in the mainstream classroom with their peers without SEN. This occurs despite the fact that a core element of inclusive education is sharing common classrooms and school spaces, providing all students with the opportunity to learn alongside one another (13). The barriers examined below likely explain this situation at least in part.

Also consistently with many other studies (2, 8, 39, 40, 45), it was found that students with disabilities/SEN participate significantly less frequently in “Getting together with peers outside of class”, that is, informal school situations such as playing at recess or engaging in social interactions between classes or during lunch. Peer relationships do not appear to be the reason; in fact, parents perceived them as a facilitator. A plausible explanation is that, when asked whether relationships with classmates helped or hindered their child’s participation at school, parents focused their answers on academic activities and paid less attention to social ones. However, the latter are an essential part of school life (9, 10), contribute to students’ growth, learning, and well-being (7, 14, 19, 21, 22), and provide a sense of belonging—a key element of quality participation (24). For this reason, future studies should further examine the effect of peer relationships on the participation of students with disabilities/SEN both in classroom-based activities (e.g., group work) and in less structured school situations (e.g., friendships, play).

Beyond participating less frequently, this study suggests that students with disabilities/SEN are significantly less engaged across all school activities in primary education and, at the secondary level, in “Special roles at school” and “Getting together with peers outside of class”. This finding is even more concerning, since lack of engagement in school activities is detrimental to academic achievement (7), career options and occupational attainment (11, 16, 18), social involvement in young adulthood (11, 18), well-being (19, 47), quality of life (22), and inclusion (9).

Given these results, it is not surprising that a significantly higher percentage of parents of students with disabilities/SEN report a desire for changes in their child’s participation across all school activities. To address this situation, recent studies have sought to identify contextual factors that help or hinder school participation, concluding that environmental characteristics are more relevant than personal ones (9, 24, 33, 56).

Accordingly, our second objective focused on identifying environmental facilitators and barriers for school participation. On the one hand, parents report that various cognitive, behavioral, social, and physical demands of school activities constitute a barrier to the participation of students with disabilities/SEN, which is consistent with prior research (31, 37). In order to bridge the gap between students’ characteristics and these demands, diverse authors have suggested strategies (e.g., 42). For example, to address cognitive demands, creating accessible school environments is useful—such as applying UDL principles and using easy-to-read materials and AACs (33). To manage behavioral demands, evidence supports the benefits of positive behavioral interventions and offering opportunities for advocacy, choice-making, and preference expression (5, 6). Social demands can be addressed by fostering positive peer connections and ensuring that extracurricular activities are open to all (31, 51, 52). Furthermore, providing access to technology or adapted transportation can help overcome physical demands.

Unfortunately, a substantial proportion of parents in this study report that some of the above-cited resources are not available in the schools their children attend. Although the unavailable resources most frequently cited are not specifically educational but rather family-related (money) or linked to accessibility (adapted transportation), parents highlight a range of unmet needs associated with school-related factors: information about programs, services (e.g., psycho-pedagogical counselling, speech therapy, physiotherapy), activities (e.g. inclusive extracurricular programs), supplies (e.g. technical aids, easy-to-read materials), support services tailored to students’ needs and interests (e.g. AACs, UDL, educational assistants/aides), and school policies and procedures (e.g. inclusive principles). Given that, in Spain, many of these resources are formally available in schools, parents may still regard them as insufficient or not sufficiently individualized (adapted) to their children’s characteristics, needs, and interests, and therefore incapable of ensuring their full participation in school life. This should not be overlooked, as parent perceptions of environmental supports in social contexts such as school represent a significant co-predictive factor of children’s quality of life (61).

On the other hand, parents report that the following factors facilitate their children with disabilities/SEN participation at school: teachers’ and staff’s attitudes and relationship with peers, safety conditions, physical layout, and sensory qualities. These findings are consistent with those obtained in numerous previous studies cited in the systematic review by Maciver et al. (9). Therefore, considering the International Classification of Functioning (ICF) typology of barriers —physical, social, and attitudinal— (1), it appears that the main obstacles encountered are primarily social in nature, while physical and attitudinal factors not only do not constitute a barrier but actually act as facilitators for school participation.

It is worth noting that parents demand more support services tailored to their children’s needs and interests, as well as school policies and procedures governed by inclusive and UDL principles. Both the individualization of supports and services and the implementation of inclusive principles and practices require adequate teacher training (34). In this study, 22.2% of parents rated teachers’ preparation to ensure the participation of diverse students as poor or very poor, while 23.2% considered school-level plans (i.e., school internal regulations, organization and provision of supports, coexistence plans, etc.) to be minimally or not at all inclusive.

The provision of individualized supports and services also demands new models of teacher collaboration (33). Research on teachers’ working cultures has shown that collegial support is crucial for facilitating school-aged children’s participation (62). A renewed culture of collaborative practice could help mitigate the resource insufficiencies reported by parents (14, 33, 34).

Moreover, modifying the school environment to foster children’s participation also requires collaboration with families and the children with disabilities themselves (14). It may be time to take a step further and consider “participation as a means” in pediatric rehabilitation and special education interventions (63, 64). Achieving enduring participation outcomes through participation in the intervention process necessitates creating a learning experience in which children and families are actively engaged at every stage. In pediatric rehabilitation, collaborative models already exist, supported by evidence of their feasibility and validity (28). These models could be adapted for use in school settings from early educational stages onward.

The present study contributes to the development of such collaborative models by providing a comprehensive assessment of parents’ perspectives on their children’s school participation. A key limitation, however, is the exclusive reliance on parents’ reports, which may differ from children’s and youth’s self-perceptions and may be less accurate when referring to older students, whose school experiences are less directly observable by their families. Consequently, future research should complement parental reports with student-reported measures. To address this gap, the authors are currently developing the *School Participation Questionnaire* to assess school participation directly from the perspective of students with and without disabilities.

Another limitation is that the relatively high educational level of participants may not be representative of the general population. In addition, two-parent families with higher education levels often have higher incomes and may provide more support to their children, meaning their responses may not reflect the experiences of families with lower education or income. Future research should therefore encompass families with diverse socio-economic backgrounds and examine how participation and environmental factors vary according to other relevant child, family, and school characteristics (e.g., disability severity, school type, or regional context).

A further consideration is the heterogeneity of the disability/SEN group. Students with disabilities and SEN may have condition-specific participation profiles and environmental requirements. However, our primary aim was to identify modifiable school environmental barriers and facilitators aligned with inclusive-education priorities. Additionally, subgroup analyses by disability type were not feasible because the uneven distribution of diagnostic subgroups limited statistical power and comparability. Future studies with larger and more balanced diagnostic subsamples should examine condition-specific participation patterns and environmental needs.

Finally, the cross-sectional nature of the study does not allow causal inferences regarding relationships between environmental factors and participation outcomes.

In conclusion, students with disabilities and other SEN participate less often and with lower involvement —especially in classroom activities and informal school interactions— thereby compromising school inclusion. This participation profile could be explained by the presence of environmental barriers reported by families: both a mismatch between the demands of activities and supports, and a limited availability of resources and services.

Therefore, the results of this study support the need to refocus efforts to improve educational quality and school inclusion by: a) delivering specialized support within general classrooms (minimizing pull-outs), b) including UDL to moderate cognitive and behavioral demands, c) ensuring the availability and individualization of supports, services, and assistive technologies (including access facilitators such as adapted transport), d) adopting participation and engagement —both academic and social— as usual quality indicators in school planning and review, e) improving targeted teacher training, and f) facilitating collegial collaboration. These guidelines align with the Supports–Quality of Life action framework for inclusive schooling, which links rights-based access to individualized supports and context redesign to advance participation, learning and development (65).

## Data Availability

The raw data supporting the conclusions of this article will be made available by the authors, without undue reservation.

## References

[1] World Health Organization . International classification of functioning, disability, and health. Geneva, Switzerland: WHO (2001).

[2] Anaby C Law M Coster W Bedell G Khetani M Avery L . The mediating role of the environment in explaining participation of children and youth with and without disabilities across home, school, and community. Arch Phys Med Rehabil. (2014) 95:908–17. doi: 10.1016/j.apmr.2014.01.005, PMID: 24468018

[3] Augustine L Lygnegård F Granlund M . Trajectories of participation, mental health, and mental health problems in adolescents with self-reported neurodevelopmental disorders’. Disabil. Rehabil. (2022) 44:1595–608. doi: 10.1080/09638288.2021.1955304, PMID: 34353177

[4] Şahin S Kaya O Kösea B Kar K . Investigation on participation, supports and barriers of children with specific learning disabilities. Res Dev Disabil. (2020) 101:103639. doi: 10.1016/j.ridd.2020.103639, PMID: 32259721

[5] Gorter JW Stewart D Woodbury-Smith M . Youth in transition: care, health and development. Child Care Health Dev. (2011) 37:757–63. doi: 10.1111/j.1365-2214.2011.01336.x, PMID: 22007974

[6] Shikako-Thomas K Kolehmainen N Ketelaar M Bult M Law M . Promoting leisure participation as part of health and well-being in children and youth with cerebral palsy. J Child Neurol. (2014) 29:1125–33. doi: 10.1177/0883073814533422, PMID: 24907136

[7] Carlberg L Granlund M . Achievement and participation in schools for young adolescents with self-reported neuropsychiatric disabilities: A cross-sectional study from the southern part of Sweden. Scand J Public Health. (2018) 47:199–206. doi: 10.1177/1403494818788415, PMID: 30070167

[8] Imms C Granlund M Wilson B Steenbergen B Rosenbaum P Gordon AM . Participation, both a means and an end: a conceptual analysis of processes and outcomes in childhood disability. Dev Med Child Neurol. (2017) 59:16–25. doi: 10.1111/dmcn.13237, PMID: 27640996

[9] Maciver D Rutherford M Arakelyan S Kramer JM Richmond J Todorova L . Participation of children with disabilities in school: A realist systematic review of psychosocial and environmental factors. PLoS One. (2019) 14:e0210511. doi: 10.1371/journal.pone.0210511, PMID: 30695082 PMC6350972

[10] Maciver D Tyagi V Kramer J Richmond L Todorova D Romero-Ayuso D . Development, psychometrics and feasibility of the School Participation Questionnaire: A teacher measure of participation related constructs. Res Dev Disabil. (2020) 106:103766. doi: 10.1016/j.ridd.2020.103766, PMID: 32961517

[11] Barnett T Tollit M Ratnapalan S Sawyer SM Kelaher M . Education support services for improving school engagement and academic performance of children and adolescents with a chronic health condition. Cochrane Database Syst Rev. (2023) 2:CD011538. doi: 10.1002/14651858.cd011538.pub2, PMID: 36752365 PMC9907052

[12] Specht JA King GA Servais M Kertoy M Spencer T . School roles: A way to investigate participation. Except. Educ Int. (2011) 21:2–14. doi: 10.5206/eei.v21i1.7666, PMID: 39985557

[13] Sandoval M Waitoller F . Broadening the notion of participation in inclusive education: A social justice approach. Rev Esp. Discap. (2022) 10:21–33. doi: 10.5569/2340-5104.10.02.02

[14] Kinnunen A Holopainen L . Exploring participative environments of children with learning and physical disabilities: Perspectives from parents and practitioners. Disabilities. (2025) 5:27. doi: 10.3390/disabilities5010027, PMID: 41725453

[15] Mamas C Bjorklund P Daly AJ Moukarzel S . Friendship and support networks among students with disabilities in middle school. Int J Educ Res. (2020) 103:101608. doi: 10.1016/j.ijer.2020.101608, PMID: 41869561

[16] Abbott-Chapman J Martin K Ollington N Venn A Dwyer T Gall S . The longitudinal association of childhood school engagement with adult educational and occupational achievement: findings from an Australian national study. Br Educ Res J. (2014) 40:102–20. doi: 10.1002/berj.3031, PMID: 41859965

[17] Guyard A Fauconnier J Muehlan H Cytera C Markwart H Himmelmann K . Differences in participation between young adults with cerebral palsy and their peers: A cross-sectional multicentre European study. Disability Health J. (2024) 17:101554. doi: 10.1016/j.dhjo.2023.101554, PMID: 38129262

[18] Kaelin VC Anaby D Werler MM Khetani MA . School participation among young people with craniofacial microsomia and other childhood-onset disabilities. Dev Med Child Neurol. (2024) 66:939–47. doi: 10.1111/dmcn.15628, PMID: 37138446 PMC10622330

[19] Anderson DL Graham AP Simmons C Thomas NP . Positive links between student participation, recognition and wellbeing at school. Int J Educ Res. (2022) 111:101896. doi: 10.1016/j.ijer.2021.101896, PMID: 41869561

[20] di Lieto MC Matteucci E Martinelli A Beani E Menici V Martini G . Impact of social participation, motor, and cognitive functioning on quality of life in children with Cerebral Palsy. Res Dev Disabil. (2025) 161:105004. doi: 10.1016/j.ridd.2025.105004, PMID: 40187227

[21] Presumido LM Silva ML Braccialli AC Sankako AN de Cássia R . Impact of school participation on quality of life of Brazilian children with cerebral palsy. Int J Disabil. Hum Dev. (2016) 15:23–7. doi: 10.1515/ijdhd-2014-0024, PMID: 41717541

[22] Shiozu H Kimura D Iwanaga R Kurasawa S . Participation as a predictor of quality of life among Japanese children with neurodevelopmental disorders analyzed using a machine learning algorithm. Children. (2024) 11:603. doi: 10.3390/children11050603, PMID: 38790598 PMC11119913

[23] Anaby D Avery L Gorter JW Levin MF Teplicky R Turner L . Improving body functions through participation in community activities among young people with physical disabilities. Dev Med Child Neurol. (2020) 62:640–6. doi: 10.1111/dmcn.14382, PMID: 31670397

[24] Bruno N Richardson A Kauffeldt KD Tomasone JR Arbour-Nicitopoulos K Latimer-Cheung AE . Exploring experiential elements, strategies and outcomes of quality participation for intellectual and developmental disabilities: A systematic scoping review. J Appl Res Intellect. Disabil. (2022) 35:691–718. doi: 10.1111/jar.12982, PMID: 35174582

[25] Cerqueira A Gaspar T Botelho Guedes F Godeau E Gaspar M . Chronic conditions, school participation and quality of life of Portuguese adolescents: Highlights from the Health Behavior in School aged Children study - HBSC 2018. Child Indic. Res. (2022) 15:297–313. doi: 10.1007/s12187-021-09868-3, PMID: 41868966

[26] Rodrigues A Wiesiolek C Meireles P da Rocha GA Farias RM Ferraz KM . Functionality, school participation and quality of life of school children with cerebral palsy. Fisioter. Mov. (2020) 33:e003329. doi: 10.1590/1980-5918.033.ao29, PMID: 41821979

[27] UN General Assembly . Convention on the rights of persons with disabilities: resolution/adopted by the General Assembly (2006). Available online at: http://www.refworld.org/docid/45f973632 (Accessed October 12, 2025).

[28] Palisano RJ Chiarello LA Vänskä N Sipari S . Content validity and utility of the collaborative process for action plans to achieve children’s participation goals. Disabilities. (2022) 2:626–40. doi: 10.3390/disabilities2040045, PMID: 41725453

[29] Rosenfeld L . Participation of children with disabilities across the life course: using socio-ecological perspectives to guide research and practice. Dev Med Child Neurol. (2020) 62:406. doi: 10.1111/dmcn.14442, PMID: 31879937

[30] Committee on the Rights of Persons with Disabilities . General comment No. 4 (2016) on the right to inclusive education (CRPD/C/GC/4). Geneva, Switzerland: United Nations (2016).

[31] Jeong Y . Participation, supports, and barriers of Korean children and youth with and without disabilities in the school environment. Disabil. Rehabil. (2020) 42:1667–74. doi: 10.1080/09638288.2019.1567836, PMID: 30714425

[32] Friedmann A Altschuck N Bertmann I Karsch F Petters A de Bock F . Participation of children in three Bavarian inclusive primary schools: parent and teacher perspectives. Int J Inclusive Educ. (2022) 28:2590–606. doi: 10.1080/13603116.2022.2119289, PMID: 41858497

[33] Meuser S Piskur B Hennissen P Dolmans D . Targeting the school environment to enable participation: A scoping review. Scand J Occup. Ther. (2022) 30:298–310. doi: 10.1080/11038128.2022.2124190, PMID: 36170879

[34] Bourke-Taylor HM Cotter C Lalor A Johnson L . School success and participation for students with cerebral palsy: a qualitative study exploring multiple perspectives. Disabil. Rehabil. (2017) 40:2163–71. doi: 10.1080/09638288.2017.1327988, PMID: 28524702

[35] Houtrow A Jones J Ghandour R Strickland B Newacheck P . Participation of children with special health care needs in school and the community. Acad Pediatr. (2012) 12:326–34. doi: 10.1016/j.acap.2012.03.004, PMID: 22683160 PMC4976484

[36] Coster W Law M Bedell G Khetani M Cousins M Teplicky R . Development of the participation and environment measure for children and youth: Conceptual basis. Disabil. Rehabil. (2012) 34:238–46. doi: 10.3109/09638288.2011.603017, PMID: 21981404

[37] Kara K Kaya Kara O Kose B Doğan M Cetin SY Şahin S . School participation, supports and barriers of children with and without attention deficit hyperactivity disorder. BMJ Paediatr Open. (2025) 9:e002917. doi: 10.1136/bmjpo-2024-002917, PMID: 39971613 PMC11840899

[38] Avramidis E Avgeri G Strogilos V . Social participation and friendship quality of students with special educational needs in regular Greek primary schools. Eur J Spec. Needs Educ. (2018) 33:221–34. doi: 10.1080/08856257.2018.1424779, PMID: 41858497

[39] Egilson ST Traustadottir R . Participation of students with physical disabilities in the school environment. Am J Occup. Ther. (2009) 63:264–72. doi: 10.5014/ajot.63.3.264, PMID: 19522135

[40] Eriksson L Welander J Granlund M . Participation in everyday school activities for children with and without disabilities. J Dev Phys Disabil. (2007) 19:485–502. doi: 10.1007/s10882-007-9065-5, PMID: 41868966

[41] Lamash L Bedell G Josman N . Participation patterns of adolescents with autism spectrum disorder compared to their peers: Parents’ perspectives. Br J Occup. Ther. (2020) 83:78–87. doi: 10.1177/0308022619853518, PMID: 41836481

[42] Andrianantenaina O Baladzhanov N Germain L Schneidman L Shahin S Anaby D . Participation strategies used by young people with and without physical disabilities. OTJR: Occup. Particip. Health. (2025) 45:338–49. doi: 10.1177/15394492241280198, PMID: 39315511 PMC12130587

[43] UNESCO . Towards inclusion in education: status, trends and challenges: the UNESCO Salamanca Statement 25 years on. Paris, France: UNESCO (2020).

[44] Coster W Law M Bedell G Liljenquist K Kao Y-C Khetani M . School participation, supports and barriers of students with and without disabilities. Child Care Health Dev. (2013) 39:535–43. doi: 10.1111/cch.12046, PMID: 23763254

[45] McGarty AM Melville CA . Parental perceptions of facilitators and barriers to physical activity for children with intellectual disabilities: A mixed methods systematic review. Res Dev Disabil. (2018) 73:40–57. doi: 10.1016/j.ridd.2017.12.007, PMID: 29248807

[46] Piskur B Beurskens AJ Ketelaar M Jongmans MJ Casparie BM Smeets RJ . Daily actions, challenges, and needs among Dutch parents while supporting the participation of their child with a physical disability at home, at school, and in the community: a qualitative diary study. BMC Pediatr. (2017) 17:12. doi: 10.1186/s12887-016-0768-6, PMID: 28077123 PMC5225511

[47] Carter W Sisco L Brown L Brickham D Al-Khabbaz A . Peer interactions and academic engagement of youth with developmental disabilities in inclusive middle and high school classrooms. Am J Ment Retard. (2008) 113:479–94. doi: 10.1352/2008.113:479-494, PMID: 19127658

[48] Richardson P . The school as social context: social interaction patterns of children with physical disabilities. Am J Occup. Ther. (2002) 56:296–304. doi: 10.5014/ajot.56.3.296, PMID: 12058518

[49] Almqvist L Granlund M . Participation in school environment of children and youth with disabilities: A person-oriented approach. Scand J Psychol. (2005) 46:305–14. doi: 10.1111/j.1467-9450.2005.00460.x, PMID: 15842421

[50] de Schauwer E Van Hove G Mortier K Loots G . ‘I need help on Mondays. It’s not my day. The other days, I’m ok.’- Perspectives of Disabled Children on Inclusive Education. Children Soc. (2009) 23:99–111. doi: 10.1111/j.1099-0860.2008.00159.x, PMID: 41858021

[51] Colver A Thyen U Arnaud C Beckung E Fauconnier J Marcelli M . Association between participation in life situations of children with cerebral palsy and their physical, social, and attitudinal environment: A cross-sectional multicenter European study. Arch Phys Med Rehabil. (2012) 93:2154–64. doi: 10.1016/j.apmr.2012.07.011, PMID: 22846455 PMC3826325

[52] Mancini MC Coster WJ . Functional predictors of school participation by children with disabilities. Occup. Ther Int. (2004) 11:12–25. doi: 10.1002/oti.194, PMID: 15118768

[53] Peny-Dahlstrand M Krumlinde-Sundholm L Gosman-Hedstrom G . Patterns of participation in school-related activities and settings in children with spina bifida. Disabil. Rehabil. (2013) 35:1821–7. doi: 10.3109/09638288.2012.758319, PMID: 23350762

[54] Earde PT Praipruk A Rodpradit P Seanjumla P . Facilitators and barriers to performing activities and participation in Children with cerebral palsy: Caregivers’ perspective. Pediatr Phys Ther. (2018) 30:27–32. doi: 10.1097/PEP.0000000000000459, PMID: 29252832

[55] Anaby D Hand C Bradley L DiRezze B Forhan M DiGiacomo A . The effect of the environment on participation of children and youth with disabilities: a scoping review. Disabil. Rehabil. (2013) 35:1589–98. doi: 10.3109/09638288.2012.748840, PMID: 23350759

[56] de Oliveira TW Duque M de Campos AC . Participation of children with developmental coordination disorder: A scoping review. Child Care Health Dev. (2025) 51:e70059. doi: 10.1111/cch.70059, PMID: 40090764

[57] Real Decreto Legislativo 1/2013, de 29 de noviembre, por el que se aprueba el Texto Refundido de la Ley General de derechos de las personas con discapacidad y de su inclusión social. Madrid, España: Boletín Oficial del Estado (2013). p. 289.

[58] Ley Orgánica 3/2020, de 29 de diciembre, por la que se modifica la Ley Orgánica 2/2006, de 3 de mayo, de Educación. Madrid, España: Boletín Oficial del Estado (2020). p. 340.

[59] Chien C-W Li-Tsang CWP Cheung PPP Leung KY Lin C-Y . Development and psychometric evaluation of the Chinese version of the Participation and Environment Measure for Children and Youth. Disabil. Rehabil. (2020) 42:2204–14. doi: 10.1080/09638288.2018.1553210, PMID: 31081396

[60] Khetani M Marley J Baker M Albrecht E Bedell G Coster W . Validity of the participation and environment measure for children and youth (PEM-CY) for health impact assessment (HIA) in sustainable development projects. Disabil. Health J. (2014) 7:226–35. doi: 10.1016/j.dhjo.2013.11.003, PMID: 24680052

[61] Bjornson SE Perry A . School satisfaction predicts quality of life for children with severe developmental disabilities and their families. J Appl Res Intellect. Disabil. (2025) 38:e70013. doi: 10.1111/jar.70013, PMID: 39887788 PMC11782910

[62] Rix J Hall K Nind M Sheehy K Wearmouth J . What pedagogical approaches can effectively include children with special educational needs in mainstream classrooms? A systematic literature review. Br J Spec. Educ. (2009) 24:86–94. doi: 10.1111/j.1467-9604.2009.01404.x, PMID: 41858021

[63] Anaby D Khetani M Piskur B van der Holst M Bedell G Schakel F . Towards a paradigm shift in pediatric rehabilitation; accelerating the uptake of evidence on participation into routine clinical practice. Disabil. Rehabil. (2022) 44:1746–57. doi: 10.1080/09638288.2021.1903102, PMID: 33832391

[64] Granlund M Imms C . Participation as a means–implications for intervention reasoning. Front Rehabil. Sci. (2024) 5:1399818. doi: 10.3389/fresc.2024.1399818, PMID: 38994330 PMC11236800

[65] Amor A Fernández M Verdugo M Aza A Calvo I . Towards the fulfillment of the right to inclusive education for students with intellectual and developmental disabilities: Framework for action. Educ Sci Soc. (2021) 12:95–113. doi: 10.3280/ess1-2021oa11471

